# Dynamic Wear Modeling and Experimental Verification of Guide Cone in Passive Compliant Connectors Based on the Archard Model

**DOI:** 10.3390/polym17152091

**Published:** 2025-07-30

**Authors:** Yuanping He, Bowen Wang, Feifei Zhao, Xingfu Hong, Liang Fang, Weihao Xu, Ming Liao, Fujing Tian

**Affiliations:** 1 High Speed Aerodynamics Institute, China Aerodynamics Research and Development Center, Mianyang 621000, China; tdhyp2013@126.com (Y.H.); fangliang_26@cardc.cn (L.F.); mianyangliaom@126.com (M.L.); 18674440178@163.com (F.T.); 2 School of Mechanical Science and Engineering, Huazhong University of Science and Technology, Wuhan 430074, China; 3 College of Mechanical and Carrier Engineering, Chongqing University, Chongqing 400044, China; 15334566875@163.com; 4 Chongqing Jiangheng Technology Co., Ltd., Chongqing 400030, China

**Keywords:** passive compliant connector, automatic docking system, guide cone wear, Archard model, finite element simulation, life prediction

## Abstract

To address the wear life prediction challenge of Guide Cones in passive compliant connectors under dynamic loads within specialized equipment, this study proposes a dynamic wear modeling and life assessment method based on the improved Archard model. Through integrated theoretical modeling, finite element simulation, and experimental validation, we establish a bidirectional coupling framework analyzing dynamic contact mechanics and wear evolution. By developing phased contact state identification criteria and geometric constraints, a transient load calculation model is established, revealing dynamic load characteristics with peak contact forces reaching 206.34 N. A dynamic contact stress integration algorithm is proposed by combining Archard’s theory with ABAQUS finite element simulation and ALE adaptive meshing technology, enabling real-time iterative updates of wear morphology and contact stress. This approach constructs an exponential model correlating cumulative wear depth with docking cycles (R^2^ = 0.997). Prototype experiments demonstrate a mean absolute percentage error (MAPE) of 14.6% between simulated and measured wear depths, confirming model validity. With a critical wear threshold of 0.8 mm, the predicted service life reaches 45,270 cycles, meeting 50-year operational requirements (safety margin: 50.9%). This research provides theoretical frameworks and engineering guidelines for wear-resistant design, material selection, and life evaluation in high-reliability automatic docking systems.

## 1. Introduction

In specialized equipment experiments, unmanned automatic docking of electrical circuits, pneumatic lines, optical fibers, and other pipelines for model transport vehicles presents significant technical demands. Dynamic wear control in automatic docking systems has become a critical challenge to ensure operational safety and long-term reliability [1,2], particularly given the accelerated degradation of conventional metallic guide components under frequent docking cycles. Current docking technologies are predominantly categorized into active compliance systems and passive compliance mechanisms [3,4,5,6]. Active compliance systems utilize multi-degree-of-freedom servo motor coordination to achieve high positioning accuracy (0.1 mm), but their implementation requires complex control architectures involving over 12 synchronized sensors, resulting in substantial costs [7]. Conversely, passive compliance mechanisms employ elastic elements and multi-degree-of-freedom (multi-DOF) platforms yet face inherent limitations: intricate stiffness design requirements and accelerated Guide Cone wear induced by residual preload forces, which degrade docking precision and reliability [8]. To address these challenges, this study develops a novel passive compliant connector docking system featuring an adaptive correction mechanism between the guide rod (supply side) and Guide Cone (receiving side). This design minimizes hardware costs and simplifies control architecture while prioritizing the resolution of wear life issues caused by frequent plug-in/extraction cycles. Material innovation—specifically advanced polymer composites—represents an essential pathway to overcome these tribological constraints while maintaining passive compliance advantages.

Recent advancements in wear prediction models follow three convergent trajectories: mechanistic refinement, numerical pragmatism, and data-driven integration [9,10,11,12,13,14]. Archard’s adhesive wear theory remains foundational, establishing wear volume proportionality to load and sliding distance, and inverse proportionality to material hardness [15,16,17,18]. Subsequent developments include Suh’s delamination theory, which incorporates strain accumulation effects to elucidate subsurface crack propagation mechanisms [19], and Jacobson’s multi-abrasive statistical model, which quantifies surface roughness impacts on wear rates [20]. Numerical simulations have progressed through Kapoor’s integration of load, roughness, and elastoplastic material behavior for wear depth calculations [21]; Flašker’s fatigue wear model based on fracture mechanics [22]; and Franklin’s microstructure-sensitive predictions using crystal plasticity finite element methods [23]. Significantly, Harnafi et al. recently developed a semi-analytical 3D model specifically for plain bearings in aero-engine variable stator vane (VSV) systems, addressing combined oscillatory loads (±1°, 100 Hz) and large-angle rotations (±30°) [17]. These advances provide critical foundations for implementing polymer composites in docking systems—a high-potential solution space currently underexplored in the literature on connector wear. While notable progress has been achieved in wear modeling for mechanical components under typical conditions [24,25,26,27,28]—exemplified by Saad Mukras’ iterative Archard-based wear prediction for crank-slider mechanisms [24]—existing models assume steady-state contact conditions. This limitation renders them inadequate for analyzing transient load characteristics during guide rod/cone docking processes, where dynamic material responses under time-varying contact stresses remain unaddressed.

To resolve wear issues in passive connector docking systems caused by frequent plug-in/extraction cycles, with particular emphasis on material-driven solutions, this study employs a multidisciplinary approach to address three key challenges in dynamic wear modeling: (1) analytical derivation of time-varying contact forces through dynamic mechanical analysis during Guide Cone insertion/extraction; (2) development of a dynamic contact stress integration strategy based on Archard theory, enabling bidirectional coupling simulation of surface morphology and contact stress evolution; and (3) prototype validation demonstrating high prediction accuracy while establishing quantitative benchmarks for future polymer implementations. The proposed methodology establishes a theoretical framework for optimizing high-reliability automatic docking systems, prioritizing material innovation through polymer composites to advance the intelligent development of specialized equipment.

## 2. Design and Dynamic Contact Mechanics of Passive Compliant Connector System

### 2.1. Structural Configuration of Passive Compliant Connector

#### 2.1.1. Supply/Receiving Side Components

**Figure 1:**
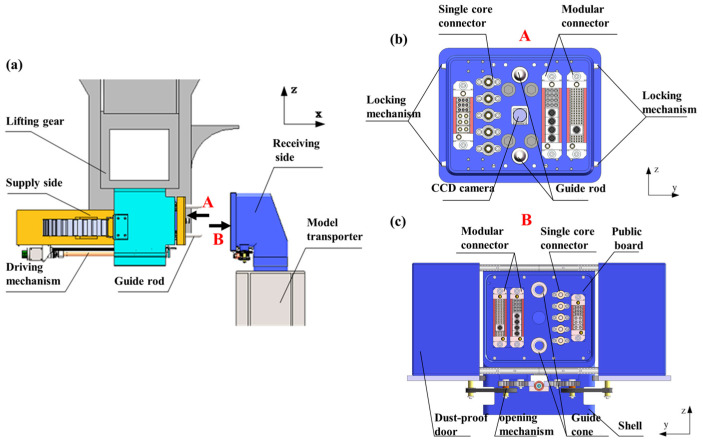
Passive compliant connector automatic docking system: (**a**) overall system, (**b**) connector female panel, and (**c**) receiving side.

As illustrated in Figure 1, the connector automatic docking system comprises three subsystems: the supply side, receiving side, and control system. The supply side integrates a propulsion mechanism, homing mechanism, adaptive mechanism, and docking female panel. The receiving side consists of a docking male panel fixed to the model transport equipment. Multifunctional interface modules—including power, communication, control, and pneumatic connectors—are embedded in both male and female panels, achieving physical–functional integrated interconnection through monolithic board-level docking design. The control system employs a dual-motor cooperative drive architecture: a primary drive motor enables active positioning along a unidirectional axis, while a guide rod–cone kinematic pair provides passive five-degree-of-freedom correction.

#### 2.1.2. Hybrid Control Strategy

**Figure 2:**
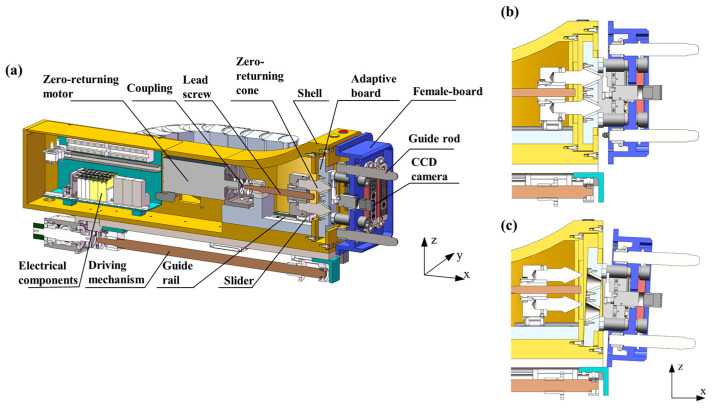
Adaptive mechanism: (**a**) overall structure, (**b**) fixed state of adaptive plate, and (**c**) anti loosening state of adaptive plate.

**Figure 3:**
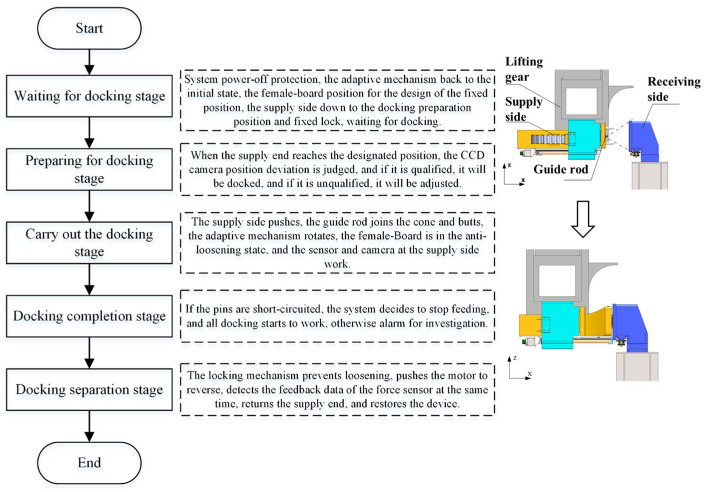
Single docking process of automatic docking system.

This configuration establishes a hybrid “active drive + passive correction” control strategy, as detailed in Figure 2. The docking process consists of two critical phases, as depicted in Figure 2b,c:

(1) The Pre-Alignment Phase: During this stage (Figure 2b), the adaptive mechanism locks the female panel to ensure precise insertion of the guide rod into the conical surface of the Guide Cone.
(2) The Compliant Adjustment Phase: Following guide pair engagement (Figure 2c), the female panel constraints are released, enabling the system to switch to compliant mode for pose adaptive correction. A dynamic force monitoring module continuously acquires contact force signals to prevent overload damage. The complete docking sequence is detailed in Figure 3.

The dual-motor cooperative control system executes distinct functions:

(1) The primary drive motor achieves positioning accuracy along the docking axis.
(2) The adaptive mechanism regulates homing cone displacement through motorized lead screw actuation, enabling female panel fixation/release state transitions (Figure 2a). During guide pair coupling, the system autonomously compensates for initial pose deviations via geometric constraints, ensuring reliable connector engagement under low contact forces. This design synergizes multi-DOF cooperative control with modular interface integration, enhancing positioning reliability in complex operational scenarios compared to conventional systems.

### 2.2. Phased Contact State Analysis

**Figure 4:**
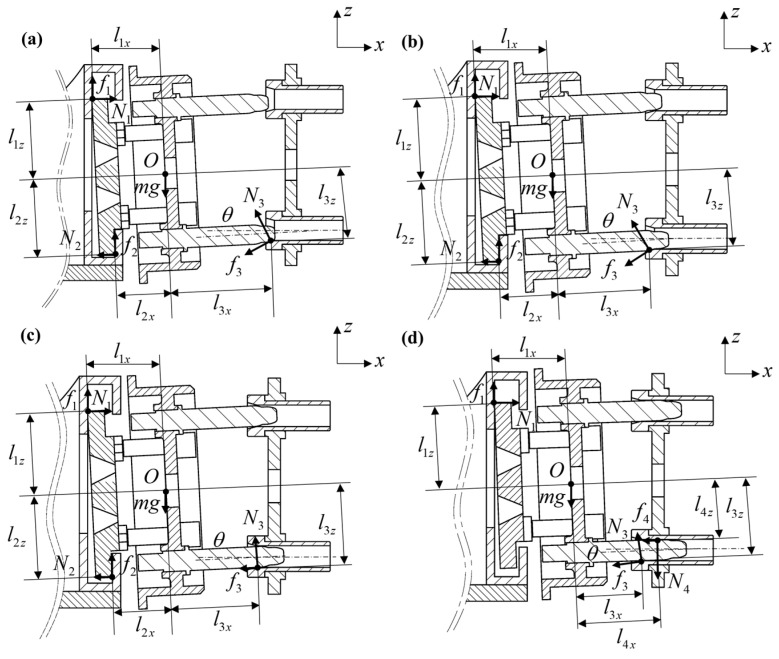
Different stages of docking system: (**a**) First Fillet Phase, (**b**) Second Fillet Phase, (**c**) Single Point Contact Phase, (**d**) Two-Point Contact Phase.

The wear depth of Guide Cones ultimately determines the docking functionality and precision of connectors [10]. To analyze the wear life of Guide Cones, a systematic investigation of their load distribution is a prerequisite. Based on the relative positioning between guide rods and cones, the docking process is categorized into four sequential phases, the First Fillet Phase, Second Fillet Phase, Single-Point Contact Phase, and Two-Point Contact Phase [8], as illustrated in Figure 4.

#### 2.2.1. Geometric Discrimination Criteria

**Figure 5:**
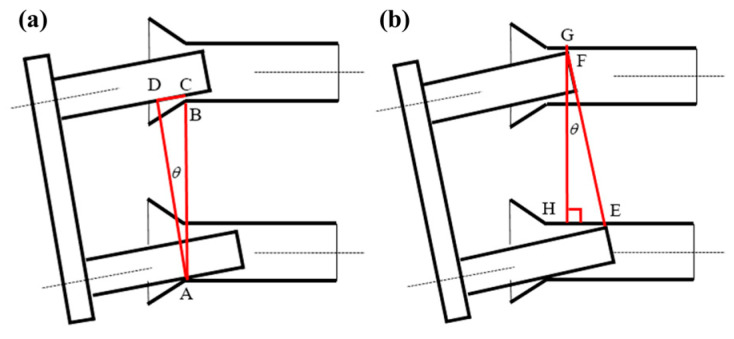
Judgment of contact state: (**a**) single-point contact; (**b**) two-point contact.

The phase terminates when stable contact is established between the cylindrical surface of the guide rod and the inner bore surface of the Guide Cone following sliding along the conical surface. A contact state identification criterion must be established to determine the coupling relationships within the guide pair (rod–cone system), as detailed in Figure 5.

Under gravitational effects, the lower guide rod initially makes contact with the lower cone (Figure 5a). Three critical points are defined: contact point A (lower cone), potential contact point B (upper cone front), and theoretical contact point C. Geometric analysis reveals that

(1) $$l_{BC}=l_{AC}-l_{AB}=l_{AD}(\frac{1}{\cos\theta }-1)$$

When *l_BC_* > 0 holds for all *θ* ∈ (0, π/2), the system maintains single-point contact with exclusive load bearing by the lower guide pair.

Transition to the Two-Point Contact Phase occurs when the lower rod’s upper surface contacts the cone’s inner surface (Figure 5b). In this configuration, contact point E on the lower guide pair and potential contact points F/G on the upper pair satisfy

(2) $$l_{GF}=l_{GH}-l_{FH}=l_{GH}(1-\cos\theta )$$

This geometric constraint (*l_GF_* > 0) ensures exclusive load bearing by the lower guide pair during two-point contact states.

#### 2.2.2. Four-Phase Dynamic Force Modeling

(1) First Fillet Phase

During the initial docking phase, the propulsion mechanism drives the female panel through a 5 mm displacement, triggering homing cone retraction. Subsequent release of adaptive plate constraints allows gravitational deflection until the first fillet of the guide rod establishes contact with the conical surface of the Guide Cone, achieving static equilibrium, as shown in Figure 4a.

Given negligible higher-order nonlinear effects from connector gravity and plug-in/extraction forces on panel tilt moments, a quasi-static model is developed. Defining initial conditions (*t* = *t*_0_ = 0 s), the static equilibrium equations for the guide pair are formulated:

(3) $$\left\{\begin{array}{l} N_{1}-N_{2}-f_{3}\cos\gamma -N_{2}\sin\gamma =0\\ f_{1}+f_{2}+N_{3}\cos\gamma -f_{2}\sin\gamma -mg=0\\ N_{1}\sin\theta l_{1x}+N_{3}\cos(\gamma -\theta )l_{3x}=N_{1}\cos\theta l_{1z}+N_{2}\sin\theta l_{2x}+N_{2}\cos\theta l_{2z}\\ +N_{3}\sin(\gamma -\theta )l_{3z}+f_{1}\cos\theta l_{1x}+f_{1}\sin\theta l_{1z}+f_{3}\sin(\gamma -\theta )l_{3x}+f_{3}\cos(\gamma -\theta )l_{3z}\\ f_{1}=\mu_{1}N_{1}\\ f_{2}=\mu_{1}N_{2}\\ f_{3}=\mu_{2}N_{3} \end{array}\right.$$

where the Guide Cone bore chamfer angle *γ* = 15°; angular displacement *θ* = 2.84°; system mass *m* = 12.2 kg; gravitational acceleration, g = 9.8 m/s^2^; friction coefficients are *μ*_1_ = 0.17 and *μ*_2_ = 0.15; and geometric dimensions are *l*_1*x*_ = 90 mm, *l*_1*z*_ = *l*_2*z*_ = 101 mm, *l*_2*x*_ = 70 mm, *l*_3*x*_ = 130.76 mm, and *l*_3*z*_ = 94.94 mm.

(2) Second Fillet Phase

To simplify computational complexity, the transition between the first and second fillet phases is assumed to be instantaneous. As shown in Figure 4b, the force equilibrium equations remain identical to Equation (3), but with modified support force coordinates: *l*_3*x*_ = 110.2 + 25sin(*γ* − *θ*); *l*_3*z*_ = 72.5 + 25cos(*γ* + *θ*).

(3) Single-Point Contact Phase

The force analysis diagram (Figure 4c) yields the following equilibrium equations:

(4) $$\left\{\begin{array}{l} N_{1}-N_{2}-f_{3}\cos\theta -N_{3}\sin\theta =0\\ f_{1}+f_{2}+N_{3}\cos\theta -f_{3}\sin\theta -mg=0\\ N_{1}\sin\theta l_{1x}+N_{3}l_{3x}+f_{2}\sin\theta l_{2x}=N_{1}\cos\theta l_{1z}+N_{2}\sin\theta l_{2x}+N_{2}\cos\theta l_{2z}\\ +f_{1}\cos\theta l_{1x}+f_{1}\sin\theta l_{1z}+f_{2}\cos\theta l_{2z}+f_{3}l_{3z} \end{array}\right.$$

The geometric parameters are defined as *l*_1*x*_ = 90 mm, *l*_1*z*_ = *l*_2*z*_ = 101 mm, *l*_2*x*_ = 70 mm, *l*_3*x*_ = (110.2 − *v*_0_(*t* − 7.25)/cos*θ*) mm, and *l*_3*z*_ = 97.5 mm.

(4) Two-Point Contact Phase

As depicted in Figure 4d, the force equilibrium equations for this phase are derived as

(5) $$\left\{\begin{array}{l} N_{1}-f_{3}\cos\theta -N_{3}\sin\theta -f_{4}=0\\ f_{1}+N_{3}\cos\theta -f_{3}\sin\theta -mg-N_{4}=0\\ N_{3}l_{3x}+N_{1}\sin\theta l_{1x}+f_{4}\sin\theta l_{4x}+f_{1}\cos\theta l_{1x}+f_{1}\sin\theta l_{1z}=\\ N_{4}\cos\theta l_{4x}+N_{4}\sin\theta l_{4z}+f_{4}\cos\theta l_{4z}+f_{3}l_{3z} \end{array}\right.$$

The geometric parameters are specified as *l*_1*x*_ = 90 mm, *l*_1*z*_ = 101 mm, *l*_3*x*_ = (80.4-*v*_0_(*t* − 12.445)/cos*θ*) mm, *l*_3*z*_ = 97.5 mm, *l*_4*x*_ = 110.2 mm, and *l*_4*z*_ = 72.5 mm.

**Figure 6:**
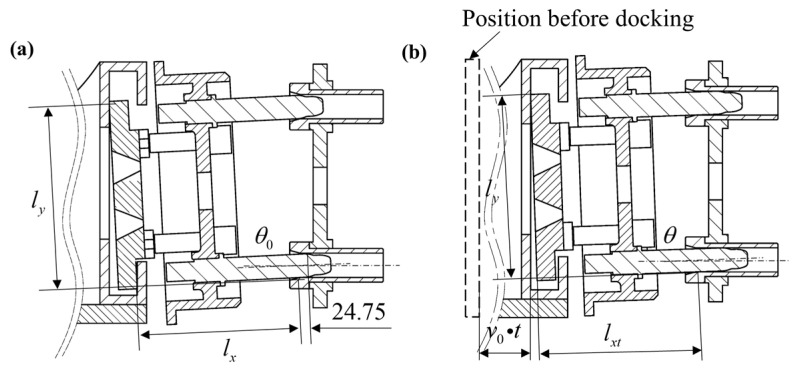
Schematic of guide rod insertion length vs. angular displacement: (**a**) pre-displacement configuration of female panel; (**b**) post-displacement state.

Figure 6a illustrates the positional relationship between the guide rod and cone at the terminal moment of the single-point contact phase, coinciding with two-point contact initiation. Figure 6b depicts the displaced configuration after supply-side movement duration, *t*, where the guide rod exhibits a deflection angle *θ*_0_ of 2.84°.

The functional relationship between the guide rod insertion depth and angular displacement at arbitrary time instants is formulated as

(6) $$\left\{\begin{array}{l} l_{y}\sin\theta_{0}+l_{x}\cos\theta_{0}-l_{y}\sin\theta -l_{xt}\cos\theta =v_{0}(t-12.445)\\ (l_{x}-l_{xt}+24.75)\sin\theta +D_{1}\cos\theta =D_{2} \end{array}\right.$$

where *l_x_* = 179.4 mm, *l_y_* = 198.5 mm, *D*_1_ = 25 mm, and *D*_2_ = 26 mm.

**Figure 7:**
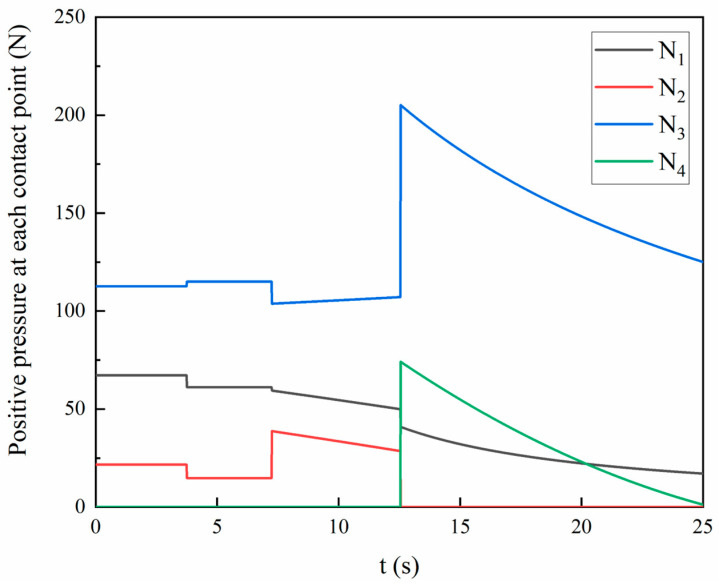
Temporal evolution of positive pressure at characteristic positions.

A systematic four-phase contact analysis of the guide pair kinematics quantifies the contact force distribution along the guide rod. Figure 7 demonstrates the temporal evolution of pressure at each contact point.

(7) $$\begin{array}{l} N_{1}=\left\{\begin{array}{l} 67.22\;\;\;\;\;\;\;0\leq t<3.75\\ 61.16\;\;\;\;\;\;3.75\leq t<7.25\\ \frac{351.6t-12534}{t-175.2}\;\;\;\;\;7.25\leq t<12.55\\ \frac{364.9}{t-3.64}\;\;\;\;\;\;\;\;\;12.55\leq t\leq 25 \end{array}\right.\;\;\;N_{2}=\left\{\begin{array}{l} 21.75\;\;\;\;\;\;\;\;\;0\leq t<3.75\\ 14.72\;\;\;\;\;\;\;\;3.75\leq t<7.25\\ \frac{351.6t-9059.1}{t-175.2}\;\;7.25\leq t<12.55\\ 0\;\;\;\;\;\;\;\;12.55\leq t\leq 25 \end{array}\right.\\ N_{3}=\left\{\begin{array}{l} 112.64\;\;\;\;\;\;\;\;\;0\leq t<3.75\\ 115.05\;\;\;\;\;\;\;\;3.75\leq t<7.25\\ \frac{17429}{175.2-t}\;\;\;\;7.25\leq t<12.55\\ \frac{3989}{t+6.887}\;\;\;\;\;\;\;12.55\leq t\leq 25 \end{array}\right.\;\;\;N_{4}=\left\{\begin{array}{l} 0\;\;\;\;\;\;\;\;\;\;\;\;0\leq t<12.55\\ 0.2t^{2}-13.37t+210.5\;12.55\leq t\leq 25 \end{array}\right. \end{array}$$

As shown in Figure 7, the calculated peak contact force of 206.34 N represents a system-specific value derived from dynamic load analysis during the docking process. This peak force originates from the inherent transient geometric constraints of the system, including the total system mass (12.2 kg), initial tilt angle (*θ* = 2.84°), friction coefficients (*μ*_1_ = 0.17 and *μ*_2_ = 0.15), and key dimensional parameters. It is important to note that the peak contact force value of 206.34 N is not universally generalizable. The force thresholds observed in practical connectors are highly dependent on specific design and material properties.

## 3. Dynamic Wear Threshold and Modified Archard Theory

### 3.1. Functional Failure Criteria

The asymmetric surface contact characteristics of the guide pair (rod–cone system) render explicit correlations between wear volume and docking cycles inherently challenging. This study adopts axial wear depth as the principal wear metric. Through geometric constraint analysis and tolerance requirement resolution, the maximum permissible wear depth *W*_hmax_ of the Guide Cone is determined.

**Figure 8:**
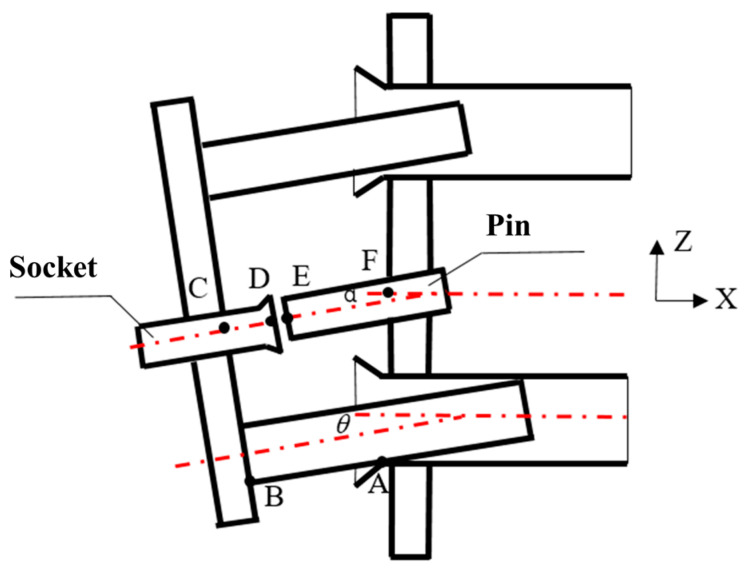
A schematic diagram of the position of the connector pin and socket end during the pre-docking stage.

The angular adaptive mechanism within the guide pair progressively corrects pose deviations at connector interfaces. As docking progresses, the progressive angular convergence between the guide rod and cone reduces pose misalignment between connector pins and sockets. Prior to docking engagement, the system must satisfy spatial pose constraints: Δ*Z* ≤ ±1 mm and Δ*Y* ≤ ±1 mm. As illustrated in Figure 8, when the wear depth reaches the critical threshold (*W*_h_ ≥ *W*_hmax_), the pose correction capability degrades below design tolerance thresholds.

Accumulated docking cycles induce monotonic growth in Guide Cone wear depth *W*_h_, accompanied by progressive degradation of pose correction capability. Upon reaching the critical threshold (*W*_hmax_), the system loses docking functionality. The determination of *W*_hmax_ requires solving the *Z*-axis positional deviation (ΔZ) between pin and socket front edges, while *X*-axis deviations can be actively compensated by the substrate. The governing geometric relationship is formulated as

(8) $$\begin{array}{l} \Delta Z=l_{DEz}=l_{EAz}-l_{DAz}=(l_{FA}-l_{EF}\sin\alpha )-(l_{CD}\sin\theta +l_{CB}\cos\theta -l_{BA}\sin\theta )\\ l_{FA}=15+0.5D_{2}+W_{\mathrm{hma}x} \end{array}$$

where *l_D__Ez_*: the *Z*-axis distance between points D and E; *l_EAz_*: the *Z*-axis distance between points E and A; *l_DAz_*: the *Z*-axis distance between points D and A; the pin extension length from male panel *l_EF_* = 52 mm; the pin depression angle α = 1.7°; the socket extension length from female panel *l_CD_* = 17 mm; the interface span between connector components *l_BA_* = 56 mm; and the *Z*-axis clearance between the connector and guide rod *l_CB_* = 15 mm.

At *t* = 16 s, the angular displacement reduces to *θ* = 1.65°. Based on connector tolerance requirements (Δ*Z* ≤ 1 mm), Equation (8) yields the theoretical maximum wear depth *W*_hmax_ = 0.91 mm. Incorporating a safety factor and machining tolerances, the design threshold is conservatively set at *W*_hmax_ = 0.8 mm.

### 3.2. Wear Depth Model Based on Archard Model

Under actual operating conditions, the pronounced time-varying characteristics of contact loads render the classical Archard model inadequate for direct characterization. To address this limitation, a modified Archard wear rate equation is formulated:

(9) $$\frac{dV}{dL}=K_{m}\frac{N(t)}{H}$$

where the wear-related sliding distance is denoted as *L* (mm); the material Brinell hardness is denoted as *H*(HB); the time-varying normal load is denoted as *N*(*t*)(N); and the dimensionless wear coefficient is denoted as *K*_m_, experimentally determined through tribological characterization of surface topography, friction regime, and lubrication conditions.

The axial wear depth d*h* is adopted as the evaluation metric, with its geometric relationship to wear volume formulated as

(10) $$dh=\frac{dV}{A}$$

where *A* represents the effective contact area (mm^2^) between the guide rod and cone.

The Guide Cone contact zone experiences time-dependent contact loads characterized by transient normal forces *N*(*t*). Consequently, the incremental wear depth can be expressed as

(11) $$dh=K_{m}\frac{v\sigma (t)}{H}dt$$

Given the angular *θ* between the mechanism’s motion direction and the guide rod’s actual sliding velocity vector, the effective sliding velocity is determined as *v* = *v*_0_/cos*θ*. The derived wear depth d*h* represents the orthogonal component relative to the sliding direction. For practical measurement alignment with the Guide Cone’s end face orientation, this depth is converted through the transformation d*h*_0_ = d*h*cos*θ*. Consequently, the modified wear depth equation becomes

(12) $$dh_{0}=dh\cos\theta =K_{m}\frac{v_{0}\sigma (t)}{H}dt$$

where *v*_0_ denotes the nominal sliding velocity along the mechanism’s primary motion axis.

Given the constant wear coefficient $K_{m}$, Brinell hardness *H*, and nominal sliding velocity *v*_0_, the cumulative wear depth of the Guide Cone is derived by integrating over the time domain [0, *t*]:

(13) $$\Delta h(t)=K_{m}\frac{v_{0}}{H}\int_{0}^{t}\sigma (t)dt$$

A discrete numerical method is implemented for iterative wear depth computation. Each docking cycle is discretized into multiple incremental steps with the time step size Δ*t*, where the contact stress σ is assumed constant within each step. The wear depth at the *j*-th time step during the *i*-th docking cycle is formulated as

(14) $$\Delta h_{j,i}=K_{m}\frac{v_{0}}{H}\sigma_{j,i}\Delta t$$

where σ*_j_*_,*i*_ are the stress values at the *j*th time step after *i* wear cycles.

To simplify calculations, equivalent contact forces during insertion/extraction are assumed. The total wear depth per complete docking cycle becomes

(15) $$\Delta h_{i}=2\sum_{j=1}^{n}\Delta h_{j,i}$$

The cumulative wear depth after m cycles is calculated as

(16) $$h(m)=\sum_{i=1}^{m}\Delta h_{i}=\frac{2K_{m}v_{0}\Delta t}{H}\sum_{i=1}^{m}\sum_{j}^{n}\sigma_{j,i}$$

The wear coefficient *K*_m_ = 3.3 × 10^−5^ is adopted based on experimental data from prior studies [29].

## 4. Finite Element Simulation Methodology Based on ABAQUS

### 4.1. Material Properties and Boundary Conditions

As summarized in Table 1, the constitutive parameters define the material behavior. Based on these properties, the Guide Cone is discretized with C3D8R hexahedral elements with a global maximum mesh size of 1 mm. A multi-tiered meshing strategy was implemented with localized refinement to 0.2 mm in critical contact zones. Mesh sensitivity analysis confirmed convergence at this configuration, demonstrating <5% variation in peak contact stress when the element size decreased below 0.2 mm while maintaining optimal computational efficiency. In ABAQUS, contact pairs are defined with the Guide Cone surface as the slave and the guide rod surface as the master, adhering to the principle of assigning higher-stiffness components as master surfaces. Contact parameters include a friction coefficient of 0.17, an augmented Lagrangian formulation, and a normal contact stiffness factor of 0.1. During iterative wear simulation, ALE adaptive meshing dynamically preserved element quality through nodal redistribution to accommodate surface morphology changes, preventing mesh distortion while ensuring solution integrity. Boundary conditions are configured as follows:

(1) Normal loads are applied along the guide rod’s contact point extension line, derived from prior contact force calculations.
(2) Guide rod motion is constrained in X/Z directions (Ux = Uz = 0) with all rotational degrees of freedom fixed (URx = URy = URz = 0).
(3) The Guide Cone is fully constrained at its base to simulate fixation on the male panel, eliminating rigid body displacements while preserving *Y*-axis mobility for docking simulation.

### 4.2. Computational Framework Implementation

**Figure 9:**
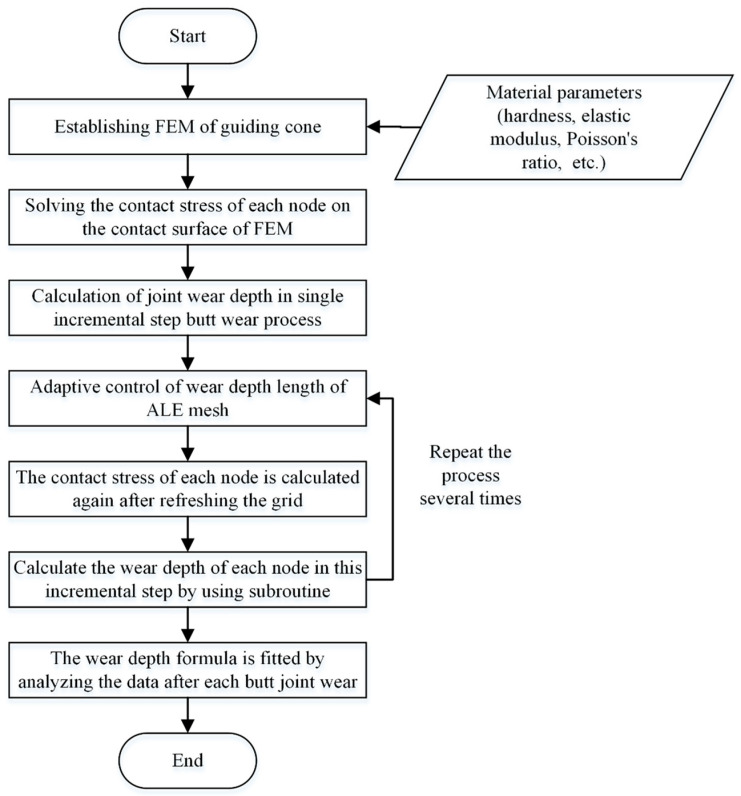
Flow chart of finite element simulation of Guide Cone wear depth.

This study establishes a multiphysics-coupled finite element model to quantitatively analyze the mapping relationship between the Guide Cone wear depth (Δ*h*) and docking cycles (*m*), with the workflow detailed in Figure 9. The methodology involved several principal phases: (1) construction of a three-dimensional model of the guide pair (rod-cone system), incorporating material constitutive relationships and hexahedral meshing with a maximum element size of 1 mm; (2) multi-step load application to extract nodal stress distributions at contact interfaces using ABAQUS/Standard; (3) implementation of Arbitrary Lagrangian–Eulerian (ALE) adaptive meshing across the Guide Cone domain, where nodal displacements induced by wear are governed by a custom Fortran subroutine (UMESHMOTION); and (4) iterative recalculation of contact stress fields following incremental mesh updates until wear depth convergence. This bidirectional coupling framework enables precise simulation of progressive wear morphology evolution through cyclic stress–wear interactions. The feedback loop between surface morphology evolution and transient contact mechanics is implemented through this UMESHMOTION subroutine, extending beyond ABAQUS’ default unidirectional wear simulation capabilities.

## 5. Experimental Validation

**Figure 10:**
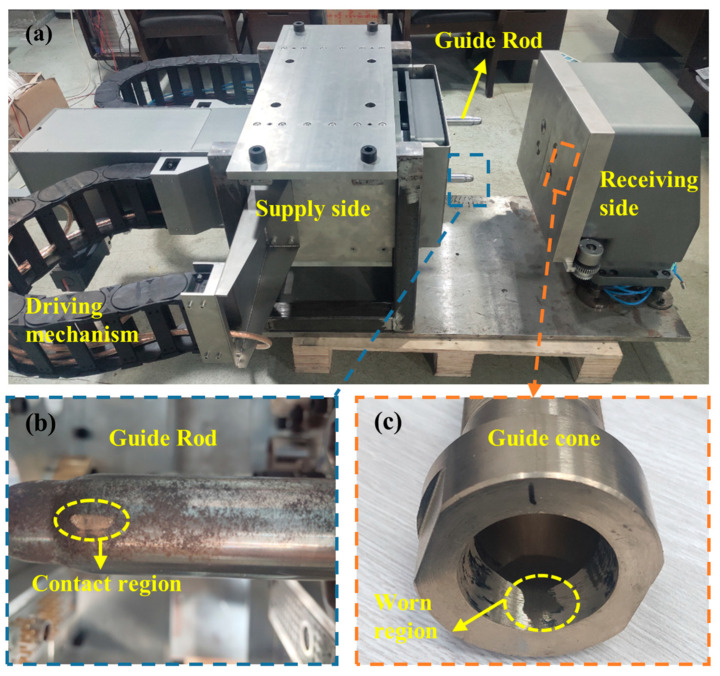
Connector automatic docking system: (**a**) prototype, (**b**) guide rod, and (**c**) guide cone.

The prototype (Figure 10) integrates a high-precision motion control system (±0.1 mm/s velocity accuracy) and force monitoring. Four Guide Cone specimens are tested: Specimens 1–3 (ZQSn5-2, HV 213.77 ± 3.26) and Specimen 4 (QSn6-6-3, HV 242.8 ± 2.15).

The experimental procedure comprises four key phases:

(1) Baseline Parameter Measurement: A digital micrometer (1 μm resolution) is used to perform quintuple axial measurements at predetermined wear zones of the Guide Cone, with triplicate circumferential sampling (120° intervals) per axial position; 3σ-filtered mean values establish baseline inner diameters.
(2) Laser displacement sensors calibrate the receiver pose to meet spatial constraints: ΔY ≤ 3 mm; ΔZ ≤ 3 mm. Gravity-induced constant contact between guide components ensures wear consistency across initial pose variations.
(3) Cyclic Docking Test: Each specimen undergoes 1500 standardized docking cycles (1.2 mm/s speed). Wear depth is quantified every 100 cycles through replicated baseline measurement protocols.
(4) Wear Mechanism Investigation: Post-test specimens are sectioned via wire EDM and analyzed using metallurgical microscopy (VHX6000, KEYENCE, Osaka, Japan) for wear morphology characterization.

## 6. Results and Discussion

### 6.1. Finite Element Simulation Results of Guide Cone Wear

**Figure 11:**
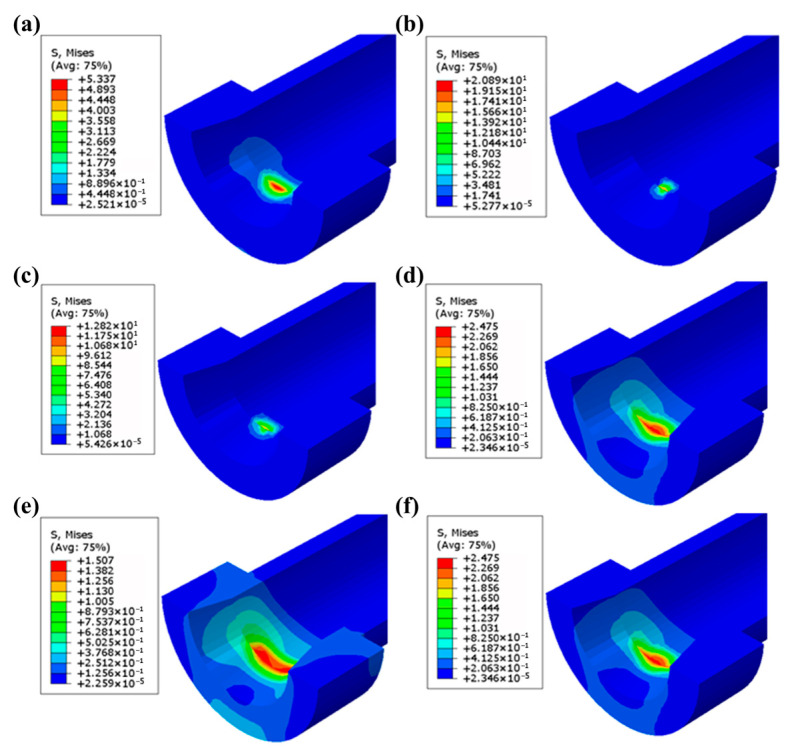
Stress nephograms of Guide Cone after (**a**) 1, (**b**) 5, (**c**) 10, (**d**) 500, (**e**) 1500, and (**f**) 7000 cycles.

The ABAQUS/Standard solver iteratively computes contact stress fields. Figure 11 displays contact stress nephograms at various docking cycles, revealing characteristic elliptical stress distributions with peak attenuation as cycles (m) increase.

**Figure 12:**
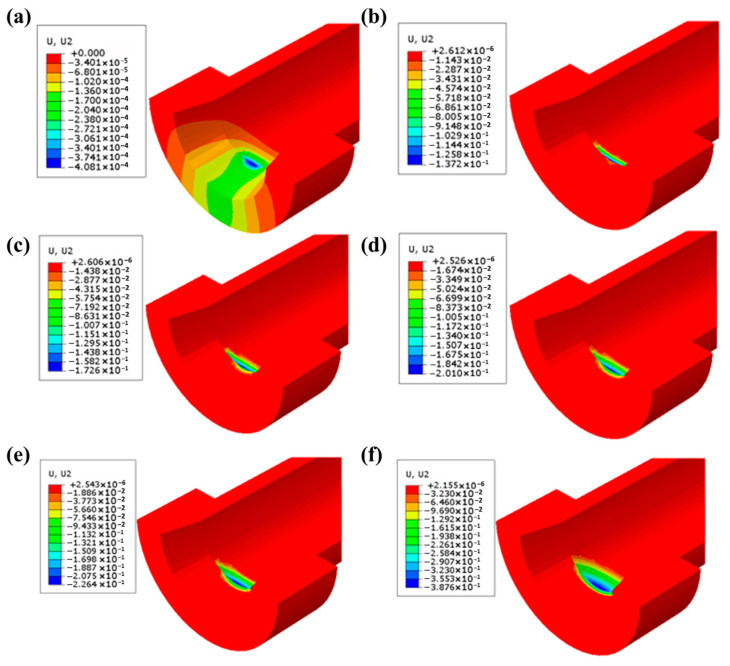
Simulated wear profiles after (**a**) 1, (**b**) 500, (**c**) 1000, (**d**) 1500, (**e**) 2000, and (**f**) 7000 cycles.

Wear depths are calculated via Equation (14) and implemented through the UMESHMOTION subroutine, driving ALE mesh adaptation. Figure 12 visualizes progressive wear accumulation, showing semi-elliptical patterns transitioning from point to area contact.

Table 2 quantifies the cumulative wear depth *W*_h_(*m*), which follows an exponential law:

(17) $$W_{h}(m)=0.01372\times m^{0.3813}-0.01774(R^{2}=0.997)$$

### 6.2. Wear Mechanism Interpretation

**Figure 13:**
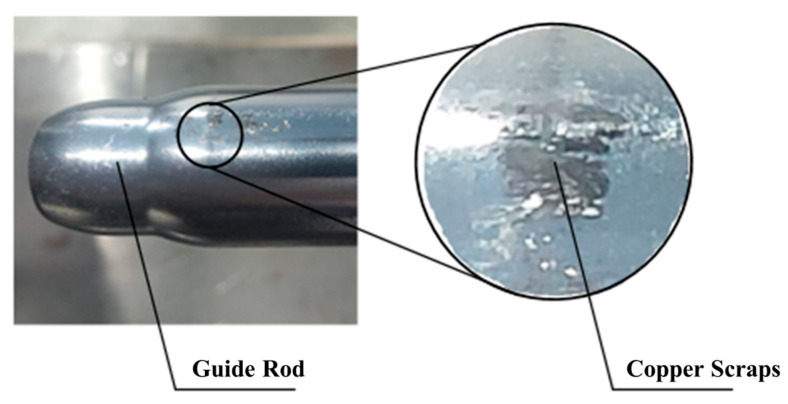
Post-wear morphology of guide rods.

As shown in Figure 13, significant copper alloy debris accumulation is observed at guide rod contact zones. This phenomenon originates from cyclic shear regeneration of adhesive junctions: plastic flow at contact interfaces generates micro-welds that undergo periodic shear failure during guide pair sliding, followed by debris re-attachment.

**Figure 14:**
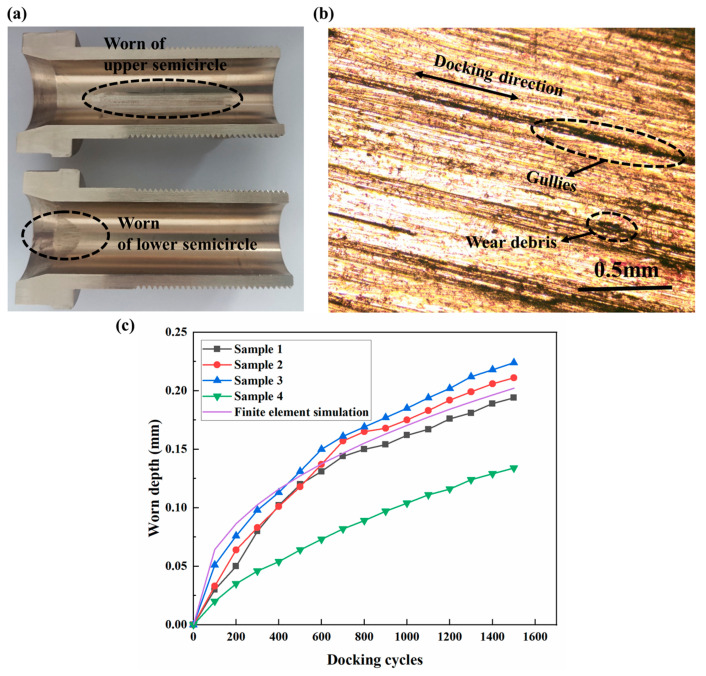
Post-wear analysis of Guide Cones: (**a**) sectioned Guide Cone, (**b**) metallographic characterization of worn surface morphology, and (**c**) cumulative wear depth vs. docking cycles for experimental specimens.

Following 1500 docking cycles, Specimens 1–3 (ZQSn5-2) exhibited comparable wear zones, while Specimen 4 demonstrated reduced wear severity. Cross-sectional analysis (Figure 14a) reveals asymmetric wear profiles:

Upper semicircle: This involves elongated gradient wear bands exhibiting left-side dominance. This asymmetry correlates with time-varying contact forces, characterized by higher left-side loads progressively decreasing toward the right.

Lower semicircle: This involves semi-elliptical wear contours matching simulated morphology (Figure 11).

Metallographic microscopy (Figure 14b) identified parallel plowing grooves and adhesive craters along the sliding direction. According to the Chinese national standard GB/T 12444-2006 [30], these morphological characteristics confirm adhesive wear as the dominant mechanism.

### 6.3. Model Accuracy Assessment

The inner diameters of Guide Cones were measured using an internal micrometer, with detailed data presented in Table 3. Figure 14c illustrates the cumulative wear depth versus docking cycles, where the simulation results (purple solid line) exhibit consistent trends with experimental data from No. 1–3. The cumulative wear depth of the Guide Cone demonstrates a monotonic increase with docking cycles, exhibiting progressive rate attenuation—an initial rapid wear phase transitions to stabilized progression. This behavior arises from evolving contact mechanics: elevated local stresses caused by limited initial contact areas gradually diminish as wear-induced contact area expansion reduces interfacial stress concentrations, resulting in corresponding wear rate decay.

Comparative wear tests reveal a 42.99% reduction in wear rates for QSn6-6-3 (HV 242.8) compared to ZQSn5-2 (HV 213.77), establishing a direct proportionality between material hardness and wear resistance. Prototype validation confirms effective parameter matching (material properties, docking velocity, and peak contact force) between experimental and operational systems, maintaining the simulation-to-test mean absolute percentage error of 14.6% (maximum 18.8%). The prediction accuracy of the proposed dynamic wear model, characterized by a mean absolute percentage error (MAPE) of 14.6% (max 18.8%) for wear depth, aligns with or marginally improves upon established benchmarks in comparable wear life studies. For instance, advanced tribological models integrating thermal–electrical–mechanical coupling for electrical connectors demonstrate qualitative validation but lack explicit error quantification, instead emphasizing trends like wear profile evolution. While supervised deep learning models for remaining useful life (RUL) prediction in industrial settings achieve higher accuracy, they require extensive labeled failure data [31]. This performance is considered acceptable in engineering contexts, as evidenced by wear life models for prostheses (e.g., knee replacements) that prioritize parametric sensitivity trends over absolute error metrics [32]. This work advances the field by providing the first experimentally validated transient connector docking wear model (14.6% MAPE) with quantitatively verified life prediction.

## 7. Model Validation and Life Prediction

### 7.1. Life Verification

The wear life assessment of Guide Cones employs the critical wear depth threshold *W*_hmax_ of 0.8 mm as the failure criterion. Substituting this value into Equation (17) yields a theoretical service life of 45,270 cycles. Considering engineering requirements of 300 operational days per year with two daily docking operations over a 50-year lifespan, and the total design demand equates to 30,000 cycles. This configuration achieves a safety margin of 50.9%, validating the system’s reliability under extended service conditions. In the current study, environmental conditions were deliberately controlled (25 °C ± 2 °C, 45% ± 5% RH, dry contact) to isolate mechanical wear mechanisms, with simulations assuming isothermal material properties. This standardized approach ensures baseline validity but limits applicability to environments where temperature fluctuations (>50 °C) reduce material hardness through thermal softening; elevated humidity (>80% RH) induces corrosive wear via oxide formation; and lubricant absence neglects protective film formation that reduces friction coefficients and contact stresses. These exclusions conservatively bias our 45,270-cycle life prediction as real-world deployments typically operate below threshold environmental severity.

### 7.2. Model Limitations and Enhancement Methodologies

#### 7.2.1. Constraints in Theoretical Framework

The present dynamic wear model, while validated for the studied passive compliant connectors, exhibits four primary constraints requiring critical acknowledgment. First, the quasi-static assumption inherently neglects inertial effects and transient dynamics during phase transitions (e.g., rod-cone impact at velocities > 1.2 mm/s), potentially underestimating peak contact stresses under high-docking-speed scenarios (>5 mm/s). This simplification arises from the force equilibrium equations (Equations (3)–(5)) excluding acceleration terms, limiting model generalizability to systems with rapid engagement kinetics. Second, friction coefficient homogenization assumes a constant value (*μ* = 0.17) despite experimental evidence of pressure- and velocity-dependent variations. This limitation obscures tribological nuances in boundary lubrication regimes, where friction may decrease nonlinearly with sliding velocity. Third, the material homogeneity postulate ignores subsurface microstructure gradients—particularly in tin–bronze alloys (ZQSn5-2) where interdendritic Sn segregation creates hardness variations. Consequently, the model cannot resolve wear mechanisms like delamination or fatigue spalling. Fourth, the synergistic interaction between material fatigue and wear constitutes a critical yet unaddressed factor, where cyclic contact stresses (peaking at 206.34 N) simultaneously drive subsurface fatigue damage and surface wear. Notwithstanding these limitations, the framework provides a foundational methodology for wear prediction in standard operating conditions (*v*_0_ ≤ 1.2 mm/s, T < 50 °C), with quantified error bounds enabling conservative life estimates.

#### 7.2.2. Pathways to Extended Docking System Service Life

To further extend the operational lifespan of docking systems beyond 45,270 cycles, four strategies can be implemented: First, material optimization through adoption of higher-hardness alloys (e.g., QSn6-6-3, HV 242.8) demonstrates a 42.99% lifespan increase. Second, tribological pair screening via standardized testing identifies material combinations with minimized wear rates. Third, a lubricant utilizing optimized formulations reduces peak contact forces, directly lowering interfacial shear stresses. Fourth, advanced polymer composites—particularly carbon fiber-reinforced PEEK and PTFE systems—offer transformative potential through their superior tribomechanical properties, namely low friction coefficients (*μ* = 0.10–0.25), exceptional wear resistance (specific wear rates ~10^−6^ mm^3^/(N·m) [33,34,35,36]), and weight reduction (40–65%), while maintaining structural integrity via enhanced fatigue resistance, dimensional stability, and creep resistance. These composites achieve performance through controlled crystallinity and an optimized filler content (15–20 wt% carbon fibers [34,35], 15–30 wt% PTFE/MoS_2_), with net-shape manufacturability via injection molding enabling complex geometries unattainable with metals [34]. Accelerated wear testing confirms significantly lower volumetric loss versus bronze under equivalent contact pressures [36]. Polymer composites could extend service life by 200% compared to metallic Guide Cones: conservative wear modeling extrapolating PEEK/40Cr pairs would achieve 90,540 cycles at equivalent loading conditions. This enhancement stems from their order-of-magnitude lower specific wear rates (approximately 10^−7^–10^−6^ mm^3^/(N·m) [37]) versus ZQSn5-2/40Cr (approximately 10^−5^ mm^3^/(N·m) [38]). Key implementation challenges include temperature-dependent crystallinity effects and anisotropic wear sensitivity to fiber orientation, necessitating future topology-optimized designs. For critical wear zones, hybrid designs incorporating metallic substrates with polymer composite inserts further extend service life while preserving structural stiffness [34].

## 8. Conclusions

To address the dynamic wear life prediction challenges of Guide Cones in passive compliant connectors, this study proposes a wear prediction methodology based on the dynamic Archard theory through systematic theoretical modeling, numerical simulation, and experimental validation. The principal conclusions are summarized as follows:

(1) The evolutionary patterns of dynamic contact loads on Guide Cones are elucidated through the establishment of phased contact state criteria and geometric constraints. By analyzing force equilibrium relationships during the four-phase engagement process, transient load characteristics are quantified with peak contact forces reaching 206.34 N, establishing a mechanical foundation for dynamic wear modeling.
(2) An iterative Archard algorithm incorporating dynamic contact stress integration is developed, creating a bidirectional wear morphology–stress coupling simulation framework. Utilizing ABAQUS/Standard with ALE adaptive meshing, discrete wear depth calculations reveal an exponential decay relationship between cumulative wear depth and docking cycles, achieving a predicted service life of 45,270 cycles for bronze alloy cone systems.
(3) Prototype testing demonstrates strong agreement between simulated and measured wear depths, with a mean absolute percentage error of 14.6%. Metallographic analysis confirms adhesive wear as the dominant mechanism, showing a 42.99% wear rate reduction in QSn6-6-3 (HV 242.8) compared to ZQSn5-2 (HV 213.77).
(4) The cumulative wear-based life assessment verifies that 45,270 cycles at the 0.8 mm critical threshold exceed 50-year requirements (30,000 cycles) with a 50.9% safety margin, outperforming conventional empirical approaches. Polymer composites emerge as the highest-potential solution for extending the service life of Guide Cones: compared to metal, the cyclic life of carbon-fiber-reinforced PEEK systems has been increased by 200%, achieved through order-of-magnitude lower wear rates (10^−7^–10^−6^ mm^3^/(N·m)). This positions polymer optimization as the pivotal strategy for next-generation docking systems. Future work will prioritize experimental validation of polymer composites, with a parallel investigation of multi-factor mechanisms (temperature-dependent crystallinity and fiber orientation effects) to unlock their full lifespan potential.

## Figures and Tables

**Table 1:** Material constitutive parameters of guiding pair.

	Material	Elastic Modulus E (GPa)	Poisson’s Ratio *v*	Vickers Hardness HV	Brinell Hardness HB
Guide rod	40Cr	211	0.3		
Guide cone	ZQSn5-2	103	0.3	213.77 ± 3.26	202.77 ± 3.26

**Table 2:** Guide pin cumulative wear depth and docking cycles.

Docking Cycles	Cumulative Wear Depth (mm)	Docking Cycles	Cumulative Wear Depth (mm)
1	4.08 × 10^−4^	1000	0.173
50	3.29 × 10^−2^	2000	0.226
100	5.32 × 10^−2^	3000	0.267
200	8.37 × 10^−2^	4000	0.303
300	0.11	5000	0.335
400	0.128	6000	0.362
500	0.137	7000	0.388

**Table 3:** Wear depth of Guide Cone.

Docking Cycles	Sample 1		Sample 2		Sample 3		Sample 4	
	Inside Diameter (mm)	Cumulative Wear Depth (mm)	Inside Diameter (mm)	Cumulative Wear Depth (mm)	Inside Diameter (mm)	Cumulative Wear Depth (mm)	Inside Diameter (mm)	Cumulative Wear Depth (mm)
0	26.031	0	26.018	0	26.034	0	26.030	0
100	26.061	0.030	26.051	0.033	26.085	0.051	26.050	0.020
200	26.081	0.050	26.082	0.064	26.11	0.076	26.065	0.035
300	26.111	0.080	26.101	0.083	26.132	0.098	26.076	0.046
400	26.133	0.102	26.119	0.101	26.147	0.113	26.084	0.054
500	26.151	0.120	26.136	0.118	26.165	0.131	26.094	0.064
600	26.161	0.131	26.155	0.137	26.184	0.150	26.103	0.073
700	26.175	0.144	26.175	0.157	26.195	0.161	26.112	0.082
800	26.181	0.150	26.183	0.165	26.203	0.169	26.119	0.089
900	26.185	0.154	26.186	0.168	26.211	0.177	26.127	0.097
1000	26.193	0.162	26.193	0.175	26.219	0.185	26.134	0.104
1100	26.198	0.167	26.201	0.183	26.228	0.194	26.141	0.111
1200	26.207	0.176	26.210	0.192	26.236	0.202	26.146	0.116
1300	26.212	0.181	26.217	0.199	26.246	0.212	26.154	0.124
1400	26.220	0.189	26.224	0.206	26.252	0.218	26.159	0.129
1500	26.225	0.194	26.229	0.211	26.258	0.224	26.164	0.134

## Data Availability

The original contributions presented in this study are included in the article. Further inquiries can be directed to the corresponding author.

## Abbreviations

The following abbreviations are used in this manuscript:

ALE	Arbitrary Lagrangian–Eulerian
MAPE	Mean Absolute Percentage Error
DOF	Degree of Freedom
VSV	Variable Stator Vane
